# Study on the Influence of Social Capital on Farmers’ Participation in Rural Domestic Sewage Treatment in Nanjing, China

**DOI:** 10.3390/ijerph17072479

**Published:** 2020-04-05

**Authors:** Rui Zhang, Huawei Zheng, Hui Zhang, Feng Hu

**Affiliations:** 1 School of Resources and Environmental Science, Nanjing Agricultural University, Nanjing 210095, China; zhangrui@njau.edu.cn (R.Z.); fenghu@njau.edu.cn (F.H.); 2 School of Humanities and Social Development, Nanjing Agricultural University, Nanjing 210095, China; 2018810093@njau.edu.cn

**Keywords:** rural domestic sewage treatment, participation behavior, factor analysis method, logistic model, social capital

## Abstract

Rural domestic sewage treatment is not only an important part of the renovation of rural human settlements, but also a major measure to revitalize those areas. In the absence of extensive participation by farmers, it is difficult to achieve desired results. From the theoretical analysis of the influence of social capital on farmers’ participation, and based on the survey data of farmers in Nanjing, Jiangsu Province, this study used a logistic model to analyze the influence of social capital and personal, family, and awareness characteristics of farmers on their participation levels. Social capital plays a significant role in promoting farmers’ participation, and the contribution of its core variables is in the following order: social norms > social trust > social networks. Among the control variables, the need for domestic sewage treatment, participation in environmental training, educational level, and participation in a village cadre significantly enhance farmers’ participation levels. Consequently, promotion of rural domestic sewage treatment should include improvement of farmers’ social trust, social norms, and social networks, to enhance social capital. Publicity and education should be reinforced, and environmental training should be carried out to improve farmers’ awareness and sense of responsibility, leading them to active participation.

## 1. Introduction

The implementation of a rural revitalization strategy is a major historical task in decisively building a well-off society and a modern socialist country in an all-round way, as well as a general starting point for China’s “agriculture, rural areas, and farmers” work in the new era [1]. Improving human settlements in rural areas and building beautiful and livable villages are an important task in implementing the strategy of rural revitalization, which is related to the fundamental well-being of most farmers [2]. Rural domestic sewage treatment (RDST) is a crucial part of the renovation of rural human settlements, and a key measure to implement the strategy of rural revitalization. In recent years, China has promoted RDST and achieved certain results, thus improving the rural ecological environment and the quality of life for farmers. Currently, rural domestic sewage treatment remains a prominent weak link in rural human settlements. Problems include: insufficient investment in environmental protection infrastructure, inadequate management and protection mechanisms, and incomplete mechanisms for farmer participation [3,4]. The level of rural domestic sewage treatment is not high. The renovation of rural human settlements with RDST as an important content is a long-term process, needs sufficient time, and extensive participation of farmers [2]. Farmers are both the subjects and beneficiaries of the renovation of rural human settlements. However, for a long time, the government has been promoting the RDST project “from top to bottom” in China. This makes all levels of governments and their functional departments become the subjects of RDST, such that that the real subjects (farmers) are excluded. The participation of farmers is relatively low, which is likely to lead to problems including the governance of the project not meeting the famers’ needs and poor management and maintenance, etc., resulting in ineffective RDST [4]. The extensive participation of farmers is the key to achieving sustainable results of RDST. Combining the "top to bottom" policies formulation and implementation with the "bottom to top" participation of farmers, improving the participation of RDST and defining the principal role of famers is important to ensure the sustainability of governance results [2]. Based on this background, it is of immense theoretical and practical significance to analyze farmers’ participation in RDST and its influencing factors.

Experts and scholars have carried out research on RDST from different perspectives, including technology selection [5,6,7,8], effect evaluation [9,10,11,12], model selection [4,13], farmer’s participation [14,15], and path optimization [16,17,18,19]. Ye Xiang [20] and Chen Shaojun et al. [21] expounded on farmers’ willingness to pay for RDST by analyzing influencing factors from the two aspects of personal and family characteristics, and proposed countermeasures and suggestions for improvement. Qiu Qiheng [22] described farmers’ willingness to cooperate in RDST, discussing the influencing factors of personal characteristics, family characteristics, and farmers’ awareness, and put forward policy suggestions. Pan Chenying [15] set forth farmers’ willingness to contribute to RDST, analyzing the influencing factors of basic situation, family situation, and personal awareness, and put forward policy suggestions to enhance willingness to contribute. Lin Wanyu [14] described the current situation of farmers’ participation in RDST, analyzed the factors influencing farmers’ participation, and proposed policy suggestions to advance long-term RDST. In general, available studies have mainly considered the external factors influencing RDST, or have been limited to the study of factors such as personal characteristics, family characteristics, or psychological awareness of farmers. China is a typical relational society [23], and social capital exerts considerable influence on economic activities, resource allocation, and individual behavior [24]. Particularly, in rural society, social capital influences information acquisition, business success, and rural governance [25], and plays a major role in farmers’ awareness and decision-making [26]. However, there are few reports on the influences on farmers’ participation in RDST. Hence, based on the social capital theory and the survey data of farmers in Nanjing, Jiangsu Province, the present study analyzed the influence of social capital on farmers’ participation in RDST, with a view to providing a reference basis for the formulation and implementation of the government’s policies on the renovation of rural human settlements.

## 2. Theoretical Bases and Research Hypotheses

Bourdieu systematically elucidated the concept of social capital for the first time, and since then, research on social capital has entered the field of vision of experts and scholars [27,28]. Bourdieu defined social capital as the emotional relationship or resource exchange that people form during social interaction, including trust, norms, and relationship networks [28,29]. Coleman defined the concept of social capital from the perspective of functionalism and systematically reviewed the emergence, maintenance, disappearance, and mechanism of social capital [30]. Putnam divided social capital into three dimensions (social trust, norms, and relationship networks), which can improve social efficiency by promoting cooperative behavior [31]. Social capital, including social trust, social norms, and social networks as the core elements, is the kernel and foundation of collective cooperative behavior [27,31,32]. According to the social capital theory, the present study selected those core elements to construct an analytical framework of the influence of social capital on farmers’ participation in RDST. Social trust can reduce the cost of social interaction, boost the efficiency of social operation, and promote the realization of social collective action. Social norms overcome the dilemma of collective action through the organic integration of short-term “altruism” and long-term “self-interest.” Social networks promote close ties among members through continuous interaction, strengthen their sense of social responsibility and awareness of resource sharing in network interactions, and enhance their sense of identity and belonging to the social community [29,31]. Social capital is an interactive and closely linked whole of these three aspects, which can affect farmers’ participation in RDST.

Social trust, the kernel discourse of social capital theory, can create a clear expectation for the future, and then foster mutually beneficial cooperation, reduce transaction costs, and lead to improvement in rural environmental governance among participants [29,33]. Among farmers, social trust is specifically manifested in the recognition of the participants’ behavior and the imitation of the behavior of the trusted farmers. Farmers’ participation in RDST can be largely affected by the behavior of surrounding farmers, especially by the behavior of trusted farmers [34]. Benign social trust can prompt tacit cooperation among farmers, promote the sharing of environmental information, and facilitate the formulation and implementation of targeted RDST measures based on environmental information. Social trust can boost smooth communication between farmers, to avoid moral hazards in the process of cooperation and maintain the cooperative order of RDST. As such, social trust helps foster the cooperative function in RDST and prompts farmers to participate in it. Accordingly, the present study proposed the following hypothesis: Social trust will have a positive influence on farmers’ participation in RDST.

Social norms are codes of conduct for people to participate in social life. These norms form reciprocal or binding mechanisms between participants to reduce costs and difficulties, and can thus improve rural environmental governance [29,33]. Reciprocity norms can strengthen the exchanges between farmers, based on integrity and trustworthiness, establish reciprocity mechanisms, and thus, elevate the cohesion of RDST. Binding norms constrain farmers’ environmental behavior through customs, ethics, and value standards. Farmers that violate the rules can bear the punishment of losing good neighborhood relationships or losing face and reputation. Therefore, social norms can help to form a binding function for RDST and externally or internally restrict the environmental behavior of farmers, prompting farmers’ participation in RDST. Accordingly, the present study proposed the following hypothesis: Social norms will have a significant positive influence on farmers’ participation in RDST.

Social networks refer to the connections formed between participants embedded in the social structure. Among the elements that make up the networks, farmers are akin to “network nodes” and become a major part of the social networks. Benign social networks can eliminate barriers to awareness, technology, and funding for farmers’ environmental governance, and therefore advance rural environmental governance [29,33]. Farmers have a strong sense of trust and belonging in a benign social network, which can result in the sharing of environmental knowledge and information, facilitate coordinated, smooth, and orderly expression of farmers’ environmental demands and interests, as well as form a “bottom-up” decision-making mechanism for RDST. On the one hand, social capital can be derived and developed continuously through the structural relationship between farmers; on the other, easy communication between farmers in social networks can foster the communication and coordination of environmental interests and further social norms and trust. Farmers form a partnership because of their interests in the same social network, which can effectively resolve the conflict of interest generated within the community. As a result, social networks are conducive to shaping the communication function in RDST, thus prompting farmers’ participation. Accordingly, the present study proposed the following hypothesis: Social networks will have a positive influence on farmers’ participation in RDST.

## 3. Data Sources and Research Methods

### 3.1. Data Sources

Nanjing is in a prosperous area in the middle of the lower reaches of the Yangtze River, southwest of Jiangsu Province, with an area of 6587.02 km^2^. In 2018, the city had authority over 11 districts, among which, six were suburbs, viz. Jiangning District, Pukou District, Qixia District, Liuhe District, Lishui District, and Gaochun District, with a permanent resident population of 8.436 million. The city’s regional gross domestic product (GDP) was 1282.04 billion CNY, and the annual per capita disposable income of rural households was 25,263 CNY [35]. The city has implemented an action plan for the prevention and control of water pollution with priorities given to water quality improvement in key sections, protection of drinking water sources, and treatment of water pollution in the Taihu Lake. The renovation of eight black and odorous riverways in non-built-up areas and 99 black and odorous ponds in rural areas have been completed, and newly discovered problematic water bodies have been reformed, thereby basically eliminating black and odorous water bodies throughout the city. The comprehensive improvement of the rural environment has been performed over a wide area, in a thorough manner, thus reinforcing efforts to increase RDST.

In the present study, through the combination of stratified sampling and random sampling, three neighborhoods in the Jiangning District, three neighborhoods in the Liuhe District, and two neighborhoods in the Lishui District were selected in March 2018. Two to three administrative villages were selected from each township, to form a total of 20 sample villages. A total of 320 questionnaires were collected, among which 302 questionnaires were effective after review. The sample farmers had the following basic characteristics: The majority of the respondents were men, accounting for 57.6%; the majority of the respondents were aged between 54 and 63 years, with those aged above 50 accounting for 78.8%; members of the Communist Party of China accounted for 9.6%, and members of village cadres accounted for 4.6%; elementary school graduates accounted for 29.5%, and middle school graduates 29.1%; the average household population was 4.8, and the average adult labor force was 3.1; the minimum annual household income was 1000 CNY, and the maximum was 900,000 CNY; the respondents with an annual household income of less than 10,000 CNY accounted for 13.2%, and those with an annual household income higher than 70,000 CNY accounted for 22.8%.

### 3.2. Variable Selection

The dependent variable of the present study was “farmers’ participation in rural domestic sewage treatment”, which was reflected in the questionnaire by questions of “Do you pay for rural domestic sewage treatment in your village?” and “Do you work for rural domestic sewage treatment in your village?”. The answer was either yes or no. If one of the answers was yes, it meant that farmers participated in rural domestic sewage treatment, and therefore the dependent variable was assigned a value of 1; otherwise the assignment was 0. 

The core independent variable was social capital, which was measured from three dimensions: Social trust, social norms, and social networks. The selected indicators of social trust were “trust in village cadres”, “trust in neighbors”, “trust in relatives”, and “trust in highly respected villagers”. The indicators of social norms selected were “times of participation in major events in the village”, “whether you have been punished or talked about for not participating in collective activities”, and “the help of building good interpersonal relationships with people involved in lending money”. The indicators of social networks selected were “the number of relatives and friends you are in frequent contact with” and “the number of monthly contacts with neighbors”.

Previous studies have revealed that the personal, family, and awareness characteristics of farmers have important effects on farmers’ participation in RDST [14,15,22]. To make the model more scientific and reasonable, control variables were selected for these three aspects. In terms of personal characteristics, age, educational level, and participation in a village cadre were selected as control variables. In terms of family characteristics, family population and annual household expenditure were selected as control variables. In terms of awareness characteristics, participation in environmental training and the degree of need for domestic sewage treatment were selected as control variables (Table 1).

### 3.3. Model Building

The main function of factor analysis is to use fewer independent factors to substantially reflect the information of the original variables and the internal relationships between the variables [36,37]. The factor analysis method was used to measure social capital to gain the scores of indicators of social trust, social norms, and social networks.

The dependent variable of the present study was farmers’ participation in RDST, which was a binary classification variable. Therefore, a logistic model was used to examine the influence of social capital on farmers’ participation in RDST [15,24]. The model was set as follows:

(1) $$\ln(\frac{p_{i}}{1-p_{i}})=\alpha_{0}+\sum \beta_{i}x_{i}+\varepsilon$$

where, $\frac{p_{i}}{1-p_{i}}$ indicates the probability of farmers participating in RDST to the probability of farmers’ not participating in RDST; i=1,2,…,n; $p_{i}$ is the probability of the *i*-th farmer participating in RDST and 1 − $p_{i}$ is the probability of the *i*-th farmer not participating in RDST; $\alpha_{0}$ is the constant term; $x_{i}$ is the independent variable (including core independent variables and control variables); $\beta_{i}$ is the partial regression coefficient; and ε, the random disturbance term.

## 4. Results and Analyses

### 4.1. Social Capital of Farmers

Before factor analysis, the credibility and validity of the questionnaire were tested [37]. The test results showed that the Cronbach’s α coefficients of the evaluation indicators of social trust, social networks, and social norms were 0.76, 0.67, and 0.68, respectively. The Cronbach α coefficients based on standardized items were 0.74, all greater than 0.65, suggesting that the questionnaire had good credibility [38]. The Kaiser-Meyer-Olkin (KMO) value reached 0.73, and the statistics of Bartlett’s test reached 679.68, which passed the significance test at a level less than 0.05, demonstrating that the data were suitable for factor analysis. Factor analysis was carried out, common factor extraction was conducted, and common factors were reviewed on the principle that eigenvalues were greater than 1, thus obtaining three common factors (Table 2).

The total variance contribution rate of common factors reached 64.75%, which shows that common factors can basically replace the overall situation of farmers’ social capital information, indicating that the factor analysis results are valid. As shown in Table 2, the social capital indicators have larger loads on factors 1, 2, and 3, which are consistent with the expected results; the load coefficients of the common factors on the original variables are all above 0.5, all the original variables had no cross load on the common factors, and the original variables reflected good discriminant validity and convergent validity [37]. Therefore, the common factors can be named as social trust factor, social norm factor, and social networks factor. The scores of the three common factors F1, F2, and F3 were calculated based on the factor score coefficient matrix and the variance contribution rate of the common factors. The mean of social trust was 3.43 and the standard deviation was 0.74; the mean of social norms was 2.03 and the standard deviation was 0.81; and the mean of social networks was 2.71 and the standard deviation was 0.85.

### 4.2. Regression Analysis Results

A logistic model was used to analyze the influence of social capital on RDST. The first regression analysis introduced the control variables (personal characteristics, family characteristics, and awareness characteristics of farmers) into the model to obtain a benchmark model, namely Model 1. Based on the benchmark model, the second regression analysis introduced the core independent variable of social capital (social trust, social norms, and social networks) into the model to obtain Model 2 (Table 3). Generally, the chi-squared test values of Model 1 and Model 2 reached a significance level of 1%, indicating that these models are effective. It should be noted that Model 2 has stronger explanatory power owing to its inclusion of the variable of social capital that the present study focuses on. Therefore, the following analysis is mainly based on the estimation results of Model 2.

#### 4.2.1. Core Independent Variable

As can be seen from Model 2, variables such as social trust, social norms, and social networks have passed the significance test. Social trust and social networks have an influence on farmer’s participation at a significance level of 5%, and the influence of social norms on farmers’ participation passes the significance test at the level of 1%. Social capital has an enormous influence on farmers’ participation in RDST, therefore enhancing social capital can promote farmers’ participation.

Social trust exerts a positive influence on farmers’ participation in RDST, which is consistent with the research hypothesis. Compared with farmers having a relatively low level of social trust, those with a high level of social trust are more likely to participate in RDST. Elevating the social trust of farmers can promote their participation in RDST. Social trust is the adhesive that gathers the strength of all aspects of society and plays a certain role in facilitating the collective environmental actions of individual members. Social trust can build up farmers’ confidence and expectation that others will take their interests into account during the exchange, thus playing the role of an internal restraint mechanism. Social trust can heighten farmers’ future expectations, enhance their willingness to cooperate, and increase their cooperative behavior. Trust and reciprocity can result in homogeneous ethics and values, as well as form a mechanism of benefit sharing and risk sharing. Such intangible forces make individuals more willing to work together for public affairs, thereby promoting farmers’ participation in RDST.

Social norms exert a positive influence on farmers’ participation behavior, which is consistent with the research hypothesis. In Model 2, the regression coefficients are: Social norms (0.691) > social trust (0.586) > social networks (0.483). Among the indicators of the three dimensions of social capital, social norms have the greatest influence on farmer’s participation. As such, rural social norms can play a binding role and have a significant positive affect on farmers’ participation in RDST. Norms stipulate what is permitted and what is not, and informal norms such as village rules, folk conventions, and folk customs can greatly encourage collective cooperation. With the acceleration of urbanization, some moral rules in rural cultures have been deconstructed, but they continue to play a role in regulating the behavior of villagers. In rural society, if members do not participate in collective activities when necessary, they will be talked about by villagers. Hence, when making behavior choices, other members need to take into consideration the pressure of public opinion in the village. There is no doubt that such moral pressure is an intangible constraint on the villagers, therefore farmers’ participation in collective affairs will increase significantly because of the guidance of norms. Moreover, there is a consistent correlation between the times of participation in major events in the village and farmers’ participation behavior. In addition, a good relationship with the community will help when the farmer needs to borrow money. Farmers become willing to interact with the community and establish good relationships so that they can benefit from such relationships and obtain needed resources in the future.

Social networks exert a positive influence on farmers’ participation behavior, which is consistent with the research hypothesis. Regarding the control of other independent variables, the probability of farmers participating in RDST increases by 62.09% for every 1 grade increase in the level of social networks. More contacts and closer relationships between farmers and their relatives and neighbors result in a greater possibility that they will participate in RDST. This may be because geographical, blood, and business relationships are embedded in the interpersonal relationship networks of China’s rural society, and the existence of the relationship network structure has laid a solid foundation for resource mobilization. Though such bonding effects have gradually weakened with the input of foreign values, it continues to unite farmers and increase their participation. The increase in the frequency of interaction between farmers with common interests can enhance their social capital to a certain extent and raise the likelihood of successful cooperation. “High network intensity and low use cost” of social networks, a dimension of social capital, facilitates the achievement of collective action and lead farmers to actively participate in RDST.

#### 4.2.2. Control Variables

As shown in Model 2, among the variables of personal characteristics and family characteristics of farmers, the educational level and participation in a village cadre passed the significance test. The educational level affects farmers’ participation behavior at a significance level of 1%. This indicates that the farmers with a higher educational level acquire more environmental knowledge, can better understand the relationship between the environment and human health, and are therefore more willing to participate in the supply of public goods in the village. The educational level of farmers influences their awareness, and higher awareness further increases the possibility of farmers’ participation. The variable of “village cadre or not” passed the significance test at the level of 5%. If farmers participate in village cadres, they will be responsible for some village affairs, better understand the purpose of the renovation of rural human settlements, and acknowledge the importance of RDST, thus being more likely to participate in RDST.

As shown in Model 2, “the degree of need for domestic sewage treatment” passed the significance test at a level of 1%, and the regression coefficient was positive. If farmers are aware of the necessity of RDST, it indicates that they start to attach importance to human settlements, care about environmental issues, and will take action to protect the environment [39]. As a result, such awareness is highly likely to exert a remarkable influence on farmers’ participation behavior. The realization of farmer’s participation in RDST, on the one hand, should consider the role of the external environment, and on the other, relies on the role of farmers’ subjective awareness. Only with the improvement of farmers’ awareness will it be possible to further internalize their rational choices and increase their enthusiasm for participating in RDST. The positive influence of “participation in environmental training” on farmers’ participation levels passed a statistical test at a significance level of 5%. The possible explanation is that training on environmental protection can enhance farmers’ awareness of the environment, trust in policies, and understanding of the importance of environmental protection and its close relationship with themselves. Their higher level of awareness will gradually be internalized into codes of conduct and concepts, so that they will actively participate in RDST.

## 5. Conclusions and Policy Implications

### 5.1. Conclusions

Social capital exerts a considerable influence on farmers’ participation in RDST. Social trust and social networks have an influence on farmers’ participation behavior at a significance level of 5%, and social norms’ influence on farmers’ participation behavior passed the significance test at the 1% level. Social norms, social trust, and social networks play significant roles in promoting farmers’ participation in RDST (in that order of importance). Raising the level of social capital can prompt farmers’ participation in RDST.

The degree of need for domestic sewage treatment and educational level have a significantly positive influence on farmers’ participation in RDST, both of which have passed the significance test at the 1% level. Further, participation in environmental training and village cadres have a significantly positive influence on farmers’ participation in RDST, both of which passed the significance test at the level of 5%.

Overall, this study made some interesting findings. The social capital, awareness of farmers, and educational level significantly enhance farmers’ participation levels in China. We can improve the level of social capital, reinforce the publicity and education, improve the farmers’ awareness and responsibility, leading them to active participation in RDST. Furthermore, the conclusion can also be applied to rural areas in other countries.

### 5.2. Policy Suggestions

#### 5.2.1. Enhancing Social Capital

Higher social capital can promote collective action and farmers’ cooperation. Therefore, it is of vital significance to improve farmers’ social capital. Trust underlies cooperation. Only when identity and trust extend to outside of the family, can the villagers in an atomized state be capable of collective action. Hence, farmers’ trust in the villagers outside the family should be enhanced. Village organizations that truly serve the people should be set up to elevate the transparency of government affairs in the village, tighten the supervision over village affairs, and bolster the trust of villagers in village cadres. A high standard of rural culture and integrity should be tapped to arouse farmers’ general recognition of the villagers with high moral standing and foster the endogenous authority of integrity in the development of rural society. In that way, rural people of honor can serve as a bridge between grass-roots governments and farmers, play an exemplary and leading role, and enhance the cohesion and identity of the village. In this way, the villagers become active participants in the supply of public goods for environmental protection and the subject role of villagers can be brought into play.

The reward and punishment mechanism implied in social norms can play a role in shaping farmers’ behavior [27]. It is necessary to gradually establish and improve the rural social norms of reciprocity and sharing, instill the intangible resources of village rules and regulations into the behavioral norms of farmers to affect their thinking patterns and values, and gradually internalize the binding rules into villagers’ self-awareness, which can become farmers’ personal beliefs and habits [40]. This can heighten their sense of collective responsibility and foster their sense of ownership. Due to a good public opinion of the environmental and social atmospheres in rural society, village norms can produce a reassuring effect, and farmers can feel confidence in the future. Consequently, their behavior is expected to become long-term [31], and the accumulation of rural cultural and spiritual wealth can keep pace with that of material wealth.

Farmers’ decisions on the supply of public goods are not only constrained by their own conditions, but also largely influenced by the decisions of other farmers [41]. The role of social networks as the information carrier can prompt the effective propagation of policies among the villagers, so that farmers doubts can be minimized when participating in collective action and they will no longer hold the mentality of onlookers. As a result, it is imperative to broaden channels for farmers to obtain information and to increase their access to official and authoritative information. In addition, rich and diverse cultural activities should be carried out to create a platform for exchanges and cooperation between farmers, to generally improve their social networks.

#### 5.2.2. Reinforcing Publicity and Education

Effectively carrying out publicity and educational work is helpful to boost farmers’ participation. Through various media platforms, a variety of publicity and educational activities that farmers like can be carried out to bolster farmers’ awareness of RDST so that they are aware that they are not only the “victims” and “producers” of rural domestic sewage pollution, but also the subjects and beneficiaries of RDST, thus prompting farmers to become “participants” in RDST. Multiple methods can be adopted to publicize the significance of RDST in bettering rural human settlements and establishing rural ecological civilization; environmental training can be performed to raise farmers’ awareness of environmental responsibility and farmers’ confidence in bettering rural human settlements can be inspired and bolstered to alter their ideas and gradually modify their behavior regarding domestic sewage treatment. 

## Figures and Tables

**Table 1:** Variable meaning and assignment.

Variable Category	Variable Name	Variable Meaning and Assignment	Mean	Standard Deviation
Dependent variable	Farmers’ participation behavior	Participation or not: Yes = 1, No = 0	0.23	0.42
Core variables	Social trust	Factor analysis score	3.43	0.74
	Social norms	Factor analysis score	2.03	0.81
	Social network	Factor analysis score	2.71	0.85
Control variables	The degree of need for domestic sewage treatment	Not needed = 1, scarcely needed = 2, normally needed = 3, relatively needed = 4, highly needed = 5	3.77	0.90
	Participation in environmental training	Participation or not: Yes= 1, No = 0	0.12	0.33
	Age	Continuous variable	57.95	13.02
	Educational level	illiteracy = 1, elementary school =2, junior high school = 3, high school = 4, university (junior college) and above = 5	2.42	1.15
	Village cadre or not	Yes = 1, No = 0	0.05	0.21
	Annual household expenditure/10,000 yuan	Continuous variable	2.69	1.24
	Family population/person	Continuous variable	4.76	2.21

**Table 2:** Factor load matrix after rotation.

Original Variable	Factor 1	Factor 2	Factor 3
Trust in neighbors	0.81	0.15	0.22
Trust in relatives	0.74	0.11	0.18
Trust in village cadres	0.73	0.17	−0.07
Trust in highly respected villagers	0.72	0.14	−0.05
Whether you have been punished or talked about for not participating in collective activities	0.08	0.78	−0.02
The help of building good interpersonal relationships with people around to money borrowing	0.19	0.76	0.18
Times of participation in major events in the village	0.24	0.75	−0.07
The number of relatives and friends you are in frequent contact with	0.03	0.21	0.86
The number of monthly contacts with neighbors	0.12	−0.14	0.84

**Table 3:** Estimated results of influencing factors on farmer’s participation behavior.

Variable	Model 1	Model 2
The degree of need for domestic sewage treatment	0.721 (0.213) ***	0.839 (0.239) ***
Participation in environmental training	1.586 (0.464) ***	1.201 (0.505) **
Age	0.033 (0.016) **	0.028 (0.018)
Educational level	0.840 (0.206) ***	0.747 (0.214) ***
Village cadre or not	2.833 (0.879) ***	2.427 (1.090) **
Annual household expenditure	0.219 (0.137)	0.114 (0.153)
Family population	0.036 (0.074)	−0.020 (0.080)
Social trust		0.586 (0.282) **
Social norms		0.691 (0.241) ***
Social networks		0.483 (0.215) **
Constant term	−9.363 (1.646) ***	−13.535 (2.073) ***
-2Log Likelihood	239.349	216.221
Cox & Snell R^2^	0.240	0.296
Nagelkerke R^2^	0.366	0.451

Notes: *, **, and *** indicate that they have passed the significance test at the statistical levels of 10%, 5%, and 1%, respectively; the values in parentheses are standard deviations.
